# Developing a Supercourse Help Desk for India

**DOI:** 10.5195/cajgh.2013.50

**Published:** 2013-10-08

**Authors:** Mita Lovalekar, Harish K. Pemde, Babu L. Verma

**Affiliations:** 1 Department of Sports Medicine and Nutrition, University of Pittsburgh; 2 Lady Hardinge Medical College, Kalawati Saran Children’s Hospital, New Delhi, India; 3 Maharani Laxmi Bai Medical College & Hospital, Jhansi, India

**Keywords:** Help desk, Supercourse

Despite a vast majority of the world’s population residing in developing countries like India, only a small fraction of the total number of global scientific publications come from these countries. This is an unhappy situation, undesirable because of several reasons.

In order to reduce the double impact of infectious and non-communicable diseases that are common in India, health care of the country needs to be based on evidence generated from research studies. Research on diseases such as tuberculosis, malaria, dengue and their relationship with malnutrition and some other locally prevalent factors, can only be conducted locally. Research capacity to investigate such endemic diseases in India is limited due to lack of adequate access of growing professionals to training in research methods, biostatistics, epidemiological methods, logical interpretation of public health concepts and also, medical research literature in journals. It is a well-known fact that developing countries have relatively fewer research articles submitted for publications to journals, and also, have a lower acceptance rate. Whether enhancing professionals’ knowledge in research methods and biostatistics in developing countries like India, will produce increased research opportunities in terms of scientific contributions, is to be investigated and published. Such research can incorporate local cultural practices that are unique to the country.

In addition to these theoretical reasons for increasing research output in India, there is also an important practical reason. A commonly cited *mantra* in academia is publish or perish. Similar to faculty in developed countries, medical teachers in India are under pressure to publish regularly. Guidelines of the Medical Council of India (MCI) – a Government autonomous agency, responsible for regulation, control and monitoring standard of medical education in the country, issued in 1998, listed minimum qualifications for teachers in medical institutions in the country.^1^ Thus, as per the MCI, for almost all teaching specialties, minimum of 4 research publications, indexed in Index Medicus/recognized national journals, is desirable for promotion to the post of reader/associate professor. In addition, for promotion to the post of Professor in most teaching specialties, one publication in an international journal is desirable. Furthermore, MCI has made it mandatory for post graduate students to publish (sent for publication) at least one research paper before they will be eligible to appear in their final examination. These steps of MCI have increased pressure on teaching faculty to publish.

Despite pressure to publish more journal articles, the publication output from India is much lower than expected (Figure 1). In fact, during recent years, there has been an increase in productivity in India, as compared to some other South Asian countries (Figure 1). However, there is still a wide scope for further improvement. In spite of being the second most populous country in the world, India is not among the top countries in the world in terms of research productivity. India, undoubtedly, has the potential to become a research giant in public health.

A recent article published in the *International Journal of Epidemiology* reviewed status of epidemiology in the WHO South-East Asia Region (SEAR).^2^ The authors searched for peer-reviewed epidemiology publications in PubMed. Less than 5% of these articles were from SEAR, with 54.9% of the SEAR articles being only from India. The authors accepted that the number of publications might have been under-estimated by their search criteria. Further, the paper also describes a lack of adequate epidemiological and public health education, training and research in this region, though the situation is gradually improving.^2^ Pertinent to the issue of lack of training in epidemiology, a study on the quality of reporting statistics in two Indian pharmacology journals revealed that inappropriate descriptive statistics were used in 78.1% of the articles, and information about checking assumptions was missing in many articles, among other issues.^3^

A recent review report (2012) in the *Indian Journal of Public Health* on biostatistics education in India,^4^ found that presently 19 institutions offer biostatistics education of different forms in the country. Though this number has gradually progressed in recent years, such institutions are geographically unevenly distributed – mostly existent in the Southern part of the country. Authors noted that in health research, biostatistics has not been given proper importance in the country. Many times, it does happen that a group of researchers start a study without including a biostatistician on board and involve them only at a late stage. By giving a citation, authors have pointed out that amongst medical colleges in India, biostatistics is considered to be one of those subjects which students dislike the most. It is important to look for the reasons as to why motivation for learning biostatistics by medical students has been so low in the country. This could be one of the reasons for poor biostatistical quality of medical papers from India, at times. The paper also speaks of need for capacity building efforts in the country, especially in the area of research methods and biostatistics. Authors have also emphasized the need for availability of trained professionals in biostatistics to help health researchers and clinicians, and for biostatistical training including support of research methods for increasing research productivity in the country.

The Supercourse is an Open Source lecture library developed at the University of Pittsburgh Web server, and all Supercourse lectures are available without charge to any one.^5^,^6^ The Supercourse team has developed the Indian Supercourse Network as a part of the main Supercourse.^7^ The Indian Supercourse Network is a collection of lectures in epidemiology, public health and community medicine, on topics of particular interest to teachers and students in India. Currently, we have 6,700 collaborators in India, who are physicians, public health professionals, academicians and researchers. They have contributed more than 200 lectures. The Indian Supercourse allows a 2-way exchange of public health information from India to the rest of the world, and then back to India.

The question is - how can publications in public health by authors from India be increased? The solution lies in enhancing research knowledge and skills of Indian researchers along with consistent guidance to improve research capabilities. A research methods “Supercourse Help Desk” will provide the platform, needed for such support to researchers in India. The Supercourse Help Desk was launched in June 2013.^8^ We have a team of over 250 research methods experts who are ready to answer research methods and biostatistics questions. The Indian Supercourse Help Desk will connect researchers in India with research experts around the world. After a question is submitted on the Help Desk Website, the client is directed to research methods lectures and bio-statistical lectures from the Supercourse and also from other sources. After examining the background material, if the client still needs help, questions will be presented to the research methods experts. Our Help Desk is thus, designed not only to answer specific Help Desk questions, but also to build capacity and teach basic research skills. The Supercourse is uniquely poised to launch a research methods Help Desk for India due to the extensive network of Indian Supercourse faculty and local “buy in” for the Supercourse from India. Professional medical associations can play a critical role in extending these resources to their members. Such steps are likely to improve patient care, based on locally produced health research based evidences.

## Figures and Tables

**Figure 1:**
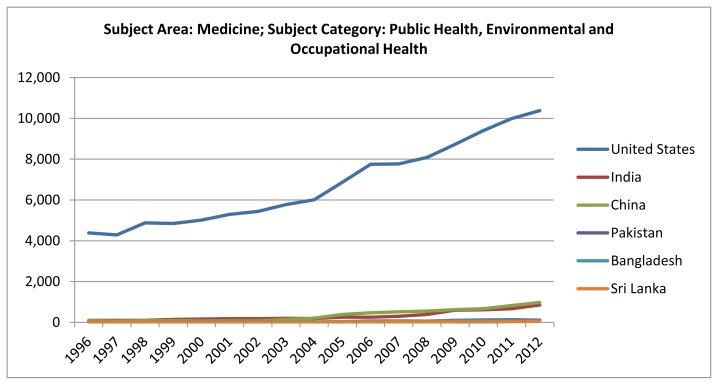
Published documents, selected countries, 1996–2012.^9^
